# Lyapunov-type inequality for a higher order dynamic equation on time scales

**DOI:** 10.1186/s40064-016-3139-8

**Published:** 2016-09-01

**Authors:** Taixiang Sun, Hongjian Xi

**Affiliations:** 1 College of Information and Statistics, Guangxi University of Finance and Economics, Nanning, 530003 China; 2 Colleges and Universities Key Laboratory of Mathematics and Its Applications, Nanning, 530004 China

**Keywords:** Lyapunov-type inequality, Dynamic equation, Time scale, 34K11, 34N05, 39A10

## Abstract

The purpose of this work is to establish a Lyapunov-type inequality for the following dynamic equation

$$S_n^\triangle (t,x(t))+u(t)x^{p}(t)=0$$

on some time scale **T** under the anti-periodic boundary conditions $S_k(a,x(a))+S_k(b,x(b))=0\, (0\le k\le n-1)$, where $S_0(t,x(t))=x(t), S_k(t,x(t))=a_k(t)S^\triangle _{k-1}(t,x(t))$ for $1\le k\le n-1$ and $S_n(t,x(t))=a_n(t)[S_{n-1}^\Delta (t,x(t))]^p$, $a_k\in C_{rd}({\mathbf{T}},(-\infty ,0)\cup (0,\infty ))\,(1\le k\le n)$ with $a_n(a)=a_n(b)$ and $u\in C_{rd}({\mathbf{T}}, {\mathbf{R}})$, *p* is the quotient of two odd positive integers and $a,b\in {\mathbf{T}}$ with $a<b$.

## Background

Lyapunov (1907) studied the following linear differential equation

1 $$x''(t) + q(t)x(t) = 0$$

and showed that if $q\in C([a, b], {\mathbf{R}})$ and $x(t)\not \equiv 0$ ($t\in [a,b]$) is a solution of (1) with $x(a) = x(b) = 0$, then the following classical Lyapunov inequality holds:

$$\int _a^b|q(t)|dt>\frac{4}{b-a},$$

Moreover, the above inequality is optimal.

Cheng (1983) investigated the following second-order difference equation

2 $$\Delta ^2x(n)+q(n)x(n+1)=0$$

and showed that if $$x(n)\not \equiv 0\, \hbox{for}\, n\in \{a,a+1,\ldots ,b\}$$ is a solution of (2) and $$x(a)=x(b)=0$$$$(a,b\in {\mathbf{Z}}$$ with $$0<a<b)$$, then $$\sum _{n=a}^{b-2}|q(n)|\ge \frac{4(b-a)}{(b-a)^2-1}$$ if $$b-a-1$$ is even and $$\sum _{n=a}^{b-2}|q(n)|\ge \frac{4}{b-a}$$ if $$b-a-1$$ is odd.

Hilger (1990) introduced the theory of time scales with one goal being the unified treatment of differential equations (the continuous case) and difference equations (the discrete case). A time scale **T** is an arbitrary nonempty closed subset of the real numbers **R**, which has the topology that it inherits from the standard topology on **R**. The two most popular examples are **R** and the integers **Z**. For the time scale calculus and some related basic concepts, we refer the readers to the books by Bohner and Peterson (2001, 2003) for further details.

Bohner et al. (2002) investigated the following Sturm–Liouville dynamic equation

3 $$x^{\Delta ^2}(t)+q(t)x^\sigma (t)=0$$

on time scale **T** under the assumptions $x(a)=x(b)=0$ ($a,b\in {\mathbf{T}}$ with $a<b$) and $q\in C_{rd}({\mathbf{T}}, (0,\infty ))$ and showed if $x(t)\not \equiv 0\,{\text{for}}\, t\in [a,b]_{\mathbf{T}}$ is a solution of (3), then

$$\int _a^bq(t)\Delta t\ge \frac{b-a}{C},$$

where $$C=\max \{(t-a)(b-t):t\in [a,b]_{\mathbf{T}}\}$$.

Wong et al. (2006) investigated the following dynamic equation

4 $$(r(t)x^\Delta (t))^{\Delta }+q(t)x^\sigma (t)=0,$$

on time scale **T** under the assumptions $x(a)=x(b)=0$ ($a,b\in {\mathbf{T}}$ with $a<b$) and $r\in C_{rd}([a,b]_{\mathbf{T}}, {\mathbf{R}})$ is monotone and $q\in C_{rd}([a,b]_{\mathbf{T}}, (0,\infty ))$, and showed that if $x(t)\not \equiv 0\,{\text{for}}\, t\in [a,b]_{\mathbf{T}}$ is a solution of (4), then

$$\int _a^b\max \{q(t),0\}\Delta t\ge \left\{ \begin{array}{ll}\frac{r(a)(b-a)}{r(b)C}, &\quad {\text{if}}\quad r\,{\text{is}}\,{\text{increasing}} ,\\ \frac{r(b)(b-a)}{r(a)C}, &\quad {\text{if}}\quad r\, {\text{is}}\,{\text{decreasing}}, \end{array}\right.$$

where $$C=\max \{(t-a)(b-t):t\in [a,b]_{\mathbf{T}}\}$$.

In this paper, we establish a Lyapunov-type inequality for the following higher order dynamic equation

5 $$S_n^\triangle (t,x(t))+u(t)x^{p}(t)=0$$

on some time scale **T** under the following anti-periodic boundary conditions

6 $$S_k(a,x(a))+S_k(b,x(b))=0\quad (0\le k\le n-1),$$

where $S_0(t,x(t))=x(t), S_k(t,x(t))=a_k(t)S^\triangle _{k-1}(t,x(t))$ for $1\le k\le n-1$ and $S_n(t,x(t))=a_n(t)[S_{n-1}^\Delta (t,x(t))]^p$, $a_k\in C_{rd}({\mathbf{T}},(-\infty ,0)\cup (0,\infty ))\,(1\le k\le n)$ with $a_n(a)=a_n(b)$ and $u\in C_{rd}({\mathbf{T}}, {\mathbf{R}})$, *p* is the quotient of two odd positive integers and $a,b\in {\mathbf{T}}$ with $a<b$.

For some other related results on Lyapunov inequality, see, for example, Çakmak (2013), He et al. (2011), Jiang and Zhou (2005), Liu and Tang (2014), Tang and Zhang (2012) and Yang et al. (2014).

## Main result and its proof

### **Lemma 1** (Bohner and Peterson 2001)

*Let *$a,b\in {\mathbf{T}}$*with *$a<b$*and *$\sum ^n_{i=1}1/p_i=1$*with *$p_i>1\,(1\le i\le n)$. *Then for any functions *$f_i\in C_{rd}([a,b]_{\mathbf{T}}, {\mathbf{R}}) \, (1\le i\le n)$, *we have*

$$\int ^b_{a}\prod _{i=1}^n|f_i(t)|\triangle t\le \prod ^n_{i=1}\left\{ \int ^b_{a}|f_i(t)|^{p_i}\triangle t\right\} ^\frac{1}{p_i}.$$

### **Lemma 2**

*Let*$$a,b\in {\mathbf{T}}$$*with*$$a<b$$. *Suppose that*$$\alpha ^j_i\in {\mathbf{R}}$$*and*$$p_i\in (1,+\infty )$$*with*$$\sum ^n_{i=1}\alpha ^j_i/p_i=\sum ^n_{i=1}1/p_i=1$$$$(1\le i\le n, 1\le j\le m)$$. *Then for any functions*$$f_j\in C_{rd}([a,b]_{\mathbf{T}},(-\infty ,0)\cup (0,\infty ))\,(1\le j\le m)$$, *we have*

$$\int ^b_{a}\prod _{j=1}^m|f_j(t)|\triangle t\le \prod _{i=1}^n\left\{ \int ^b_{a}\prod _{j=1}^m|f_j(t)|^{\alpha ^j_i}\triangle t\right\} ^\frac{1}{p_i}.$$

### *Proof*

Let $F_i(t)=(\prod _{j=1}^m|f_j(t)|^{\alpha ^j_i})^{\frac{1}{p_i}}$. By Lemma 1 we have

$$\begin{aligned} \int ^b_{a}\prod _{j=1}^m|f_j(t)|\triangle t & = \int ^b_{a}\prod _{i=1}^nF_i(t)\triangle t\\ &\le \prod _{i=1}^n\left\{ \int ^b_{a}F_i^{p_i}\triangle t\right\} ^\frac{1}{p_i}\\ &= \prod _{i=1}^n\left\{ \int ^b_{a}\prod _{j=1}^m|f_j(t)|^{\alpha ^j_i}\triangle t\right\} ^\frac{1}{p_i}. \end{aligned}$$

This completes the proof of Lemma 2. $\square$

### *Remark 3*

Let $i=j$, and $\alpha _i^i=p_i$ and $\alpha _i^j=0$ if $i\ne j$ in Lemma 2, we obtain Lemma 1.

### **Theorem 4**

*Let *$\alpha _i\in {\mathbf{R}}\,(1\le i\le n)$, $p_1=p+1$*and*$p_j\in (1,+\infty )\,(2\le j\le n)$*with*$\sum ^n_{i=1}\alpha _i/p_i=\sum ^n_{i=1}1/p_i=1$. *If * (5) *has a solution* $x(t) \not \equiv 0\,{{for}}\,t\in [a,b]_{\mathbf{T}}$*satisfying the anti-periodic boundary conditions* (6), *then*

$$\int ^b_a|u(t)|^{\frac{p+1}{p}}\Delta t\ge \frac{2^{\frac{[(n-1)p+1](p+1)}{p}}}{(b-a)^{\frac{ 1}{p}}\left[ \int ^b_a\frac{\Delta t}{|a_n(t)|^{\frac{1}{p}}}\right] ^{p+1}\prod _{i=1}^{n-1}\left\{\prod _{j=1}^n\left[\int ^b_a \frac{\Delta t}{|a_i(t)|^{\alpha _i}}\right]^{\frac{1}{p_j}}\right\}^{p+1}}.$$

### *Proof*

For any $1\le i\le n-1$, write

$$w_i=\prod _{j=1}^n\left[ \int ^b_a \frac{\Delta t}{|a_i(t)|^{\alpha _i}}\right] ^{\frac{1}{p_j}}$$

and

$$u_i=\prod _{j=1}^n\left[ \int ^b_a \frac{|S_i(t,x(t))|}{|a_i(t)|^{\alpha _j}}\Delta t\right] ^{\frac{1}{p_j}}.$$

Since *x*(*t*) satisfies $S_i(a,x(a))+S_i(b,x(b))=0\,(0\le i\le n-1)$, we know that for any $t\in [a,b]_{\mathbf{T}}$,

$$S_i(t)=S_i(a,x(a))+\int ^t_a\frac{S_{i+1}(\tau ,x(\tau ))}{a_{i+1}(\tau )}\Delta \tau =S_i(b,x(b))-\int ^b_t\frac{S_{i+1}(\tau ,x(\tau ))}{a_{i+1}(\tau )}\Delta \tau.$$

Using Lemma 2, we obtain that for $0\le i\le n-2$,

7 $$\begin{aligned} |S_i(t,x(t))|&= \frac{1}{2}|S_i(a,x(a))+\int ^t_a\frac{S_{i+1}(\tau ,x(\tau ))}{a_{i+1}(\tau )}\Delta \tau +S_i(b,x(b)) -\int ^b_t\frac{S_{i+1}(\tau ,x(\tau ))}{a_{i+1}(\tau )}\Delta \tau | \\ &\le \frac{1}{2}\int ^b_a|\frac{S_{i+1}(t,x(t))}{a_{i+1}(t)}|\Delta t\le \frac{1}{2}u_{i+1}. \end{aligned}$$

and

$$\begin{aligned}&|S_{n-1}(t,x(t))|\le \frac{1}{2}\int ^b_a\frac{|a_n(t)|^{\frac{1}{p_1}}}{|a_n(t)|^{\frac{1}{p_1}}}|S^\Delta _{n-1}(t,x(t))|\Delta t \\&\quad \le \frac{1}{2}\left[ \int ^b_a\frac{\Delta t}{|a_n(t)|^{\frac{1}{p}}}\right] ^{\frac{p}{p_1}}\left[ \int ^b_a|a_n(t)| |S^\Delta _{n-1}(t,x(t))|^{p_1}\Delta t\right] ^{\frac{1}{p_1}}\quad (t\in [a,b]_{\mathbf{T}}) \end{aligned}$$

and

$$|S_{n-1}(\sigma (t),x(\sigma (t)))| \le \frac{1}{2}\left[ \int ^b_a\frac{\Delta t}{|a_n(t)|^{\frac{1}{p}}}\right] ^{\frac{p}{p_1}}\left[ \int ^b_a|a_n(t)| |S^\Delta _{n-1}(t,x(t))|^{p_1}\Delta t\right] ^{\frac{1}{p_1}}\quad (t\in [a,b)_{\mathbf{T}}),$$

which implies

8 $$\begin{aligned}&u_i=\prod _{j=1}^n\left[ \int ^b_a \frac{|S_i(t,x(t))|}{|a_i(t)|^{\alpha _j}}\Delta t\right] ^{\frac{1}{p_j}}. \\&\quad \le \frac{1}{2}u_{i+1}w_{i}\quad (1\le i\le n-2) \end{aligned}$$

and

9 $$|S_{n-1}(t,x(t))|^{p_1}\le \frac{1}{2^{p_1}}\left[ \int ^b_a\frac{\Delta t}{|a_n(t)|^{\frac{1}{p}}}\right] ^{p}\int ^b_a|a_n(t)||S^\Delta _{n-1}(t,x(t))|^{p_1}\Delta t\quad (t\in [a,b]_{\mathbf{T}})$$

and

10 $$|S_{n-1}(\sigma (t),x(\sigma (t)))|^{p_1}\le \frac{1}{2^{p_1}}\left[ \int ^b_a\frac{\Delta t}{|a_n(t)|^{\frac{1}{p}}}\right] ^{p} \int ^b_a|a_n(t)||S^\Delta _{n-1}(t,x(t))|^{p_1}\Delta t\quad (t\in [a,b)_{\mathbf{T}}).$$

Combining (7), (8) and (9), it follows

11 $$|x(t)|\le M\equiv \frac{\prod _{i=1}^{n-1}w_i}{2^{n-1}}\left[ \int ^b_a\frac{\Delta t}{|a_n(t)|^{\frac{1}{p}}}\right] ^{\frac{p}{p_1}}\left[ \int ^b_a|a_n(t)| |S^\Delta _{n-1}(t,x(t))|^{p_1}\Delta t\right] ^{\frac{1}{p_1}}.$$

From (1), we have

$$S^\Delta _n(t,x(t))=-u(t)x(t)^{p}.$$

Thus, we obtain

12 $$S^\Delta _n(t,x(t))S^\sigma _{n-1}(t,x(t))=-u(t)x^{p}(t)S^\sigma _{n-1}(t,x(t)).$$

Integrating (12) from *a* to *b*, it follows

13 $$\int ^b_aS^\Delta _n(t,x(t))S^\sigma _{n-1}(t,x(t))\Delta t=\int ^b_a-u(t)x^{p}(t)S^\sigma _{n-1}(t,x(t))\Delta t.$$

Thus, we obtain from (10), (11) and (13) that

$$\begin{aligned}\int ^b_aa_n(t)|S^\Delta _{n-1}(t,x(t))|^{p+1}\Delta t &= \int ^b_aa_n(t)(S^\Delta _{n-1}(t,x(t)))^{p+1}\Delta t\\ &= \int ^b_a[(S_n(t,x(t))S_{n-1}(t,x(t)))^\Delta -S^\Delta _n(t,x(t))S^\sigma _{n-1}(t,x(t))]\Delta t\\ &= a_n(b)S^p_{n-1}(b,x(b))S_{n-1}(b,x(b))-a_n(a)S^p_{n-1}(a,x(a))S_{n-1}(a,x(a))\\&\quad-\,\int ^b_aS^\Delta _n(t,x(t))S^\sigma _{n-1}(t,x(t))\Delta t\\ &\le \int ^b_a|u(t)x^{p}(t)S^\sigma _{n-1}(t,x(t))|\Delta t\\ &\le M^p\int ^b_a|u(t)||S_{n-1}(\sigma (t),x(\sigma (t)))|\Delta t\\&\le M^p\left[ \int ^b_a|u(t)|^{\frac{p_1}{p}}\Delta t\right] ^{\frac{p}{p_1}}\left[ \int ^b_a|S_{n-1}(\sigma (t),x(\sigma (t)))|^{p_1}\Delta t\right] ^{\frac{1}{p_1}}\\ & \le M^p\left[ \int ^b_a|u(t)|^{\frac{p_1}{p}}\Delta t\right] ^{\frac{p}{p_1}}\frac{(b-a)^{\frac{1}{p_1}}}{2}\left[ \int ^b_a\frac{\Delta t}{|a_n(t)|^{\frac{1}{p}}}\right] ^{\frac{p}{p_1}}\left[ \int ^b_a|a_n(t)||S^\Delta _{n-1}(t,x(t))|^{p_1}\Delta t\right] ^{\frac{1}{p_1}}\\ &= \left\{ \frac{\prod _{i=1}^{n-1}w_i}{2^{n-1}}\left[ \int ^b_a\frac{\Delta t}{|a_n(t)|^{\frac{1}{p}}}\right] ^{\frac{p}{p_1}}\left[ \int ^b_a|a_n(t)| |S^\Delta _{n-1}(t,x(t))|^{p_1}\Delta t\right] ^{\frac{1}{p_1}}\right\} ^p\\&\quad\times\, \left[ \int ^b_a|u(t)|^{\frac{p_1}{p}}\Delta t\right] ^{\frac{p}{p_1}}\frac{(b-a)^{\frac{1}{p_1}}}{2}\left[ \int ^b_a\frac{\Delta t}{|a_n(t)|^{\frac{1}{p}}}\right] ^{\frac{p}{p_1}}\left[ \int ^b_a|a_n(t)||S^\Delta _{n-1}(t,x(t))|^{p_1}\Delta t\right] ^{\frac{1}{p_1}}\\ &= \frac{[\prod _{i=1}^{n-1}w_i]^p}{2^{(n-1)p+1}}(b-a)^{\frac{1}{p_1}}\left[ \int ^b_a|u(t)|^{\frac{p_1}{p}}\Delta t\right] ^{\frac{p}{p_1}}\left[ \int ^b_a\frac{\Delta t}{|a_n(t)|^{\frac{1}{p}}}\right] ^{p}\\&\times \left[ \int ^b_a|a_n(t)||S^\Delta _{n-1}(t,x(t))|^{p+1}\Delta t\right] ^{\frac{p+1}{p+1}}. \end{aligned}$$

Since $x(t)\not \equiv 0\, (t\in [a,b]_{\mathbf{T}})$, it follows from (11) that

$$\int ^b_a|a_n(t)||S^\Delta _{n-1}(t,x(t))|^{p+1}\Delta t>0.$$

Thus, we obtain

$$\int ^b_a|u(t)|^{\frac{p+1}{p}}\Delta t\ge \frac{2^{\frac{[(n-1)p+1](p+1)}{p}}}{(b-a)^{\frac{ 1}{p}}\left[ \int ^b_a\frac{\Delta t}{|a_n(t)|^{\frac{1}{p}}}\right] ^{p+1}\prod _{i=1}^{n-1}\left\{\prod _{j=1}^n\left[\int ^b_a \frac{\Delta t}{|a_i(t)|^{\alpha _i}}\right]^{\frac{1}{p_j}}\right\}^{p+1}}.$$

This completes the proof of Theorem 4.$\square$

Let $\alpha _i=1+r_ip_i\,(1\le i\le n)$ in Theorem 4, we obtain the following corollary.

### **Corollary 5**

*Let *$r_i\in {\mathbf{R}}\,(1\le i\le n)$, $p_1=p+1$*and *$p_j\in (1,+\infty )\,(2\le j\le n)$*with *$\sum ^n_{i=1}1/p_i=1$*and *$\sum ^n_{i=1}r_i=0$. *If *(5) *has a solution *$x(t) \not \equiv 0\,{\text{for}}\,t\in [a,b]_{\mathbf{T}}$*satisfying the anti-periodic boundary conditions* (6), *then*

$$\int ^b_a|u(t)|^{\frac{p+1}{p}}\Delta t\ge \frac{2^{\frac{[(n-1)p+1](p+1)}{p}}}{(b-a)^{\frac{ 1}{p}}\left[ \int ^b_a\frac{\Delta t}{|a_n(t)|^{\frac{1}{p}}}\right] ^{p+1}\prod _{i=1}^{n-1}\left\{\prod _{j=1}^n\left[\int ^b_a \frac{\Delta t}{a_i^{1+r_ip_i}(t)}\right]^{\frac{1}{p_j}}\right\}^{p+1}}.$$

*Set *$\alpha _i=1\,(1\le i\le n)$*in Theorem* 4, *we obtain the following Corollary*6.

### **Corollary 6**

*If *(5) *has a solution *$x(t) \not \equiv 0\,{\text{for}}\,t\in [a,b]_{\mathbf{T}}$*satisfying the anti-periodic boundary conditions* (6), *then*

$$\int ^b_a|u(t)|^{\frac{p+1}{p}}\Delta t\ge \frac{2^{\frac{[(n-1)p+1](p+1)}{p}}}{(b-a)^{\frac{ 1}{p}}\left[ \int ^b_a\frac{\Delta t}{|a_n(t)|^{\frac{1}{p}}}\right] ^{p+1}\prod _{i=1}^{n-1}\left[\int ^b_a \frac{\Delta t}{a_i(t)}\right]^{p+1}}.$$

## Examples and applications

### *Example 1*

Suppose that $\alpha _i\in {\mathbf{R}}\,(1\le i\le n)$, $p_1=p+1$ and $p_j\in (1,+\infty )\,(2\le j\le n)$ with $\sum ^n_{i=1}\alpha _i/p_i=\sum ^n_{i=1}1/p_i=1$. Let ${\mathbf{T}}=[-2,-1]\cup [1,\infty )$, $a_k(t)=t$ for $1\le k\le n-1$ and $a_n=t^{2m}$ for some positive integer *m*, and

$$\begin{aligned} u(t)=\left\{ \begin{array}{ll} -(2m+1)2m(p+1)/t^{p+1-2m}, &\quad {\text{if}}\quad t\not =-1,\\ \{(2m+1)^{np}-[1/2^{n-1}+\sum _{i=1}^{n-1}(2m+1)^{n-i}/2^i]^p]\}/2, &\quad {\text{if}}\quad t=-1. \end{array}\right. \end{aligned}$$

Set $x(t)=t^{2m+1}$. It is easy to check that

1.   $$S_k(t,x(t))=(2m+1)^kt^{2m+1}\,(0\le k\le n-1)$$, $$S_n(t,x(t))=(2m+1)^{np}t^{2m(p+1)}$$ and $$S^\triangle _n(t,x(t))=(2m+1)^{np}2m(p+1)t^{2m(p+1)-1}$$ for $$t\not =-1$$.
2.   $$S_0(-1,x(-1))=S_1(-1,x(-1))=-1, S_k(-1,x(-1))=-[1/2^{k-1}+\sum _{i=1}^{k-1}(2m+1)^{k-i}/2^i]$$$$(0\le k\le n-1)$$, $$S_n(-1,x(-1))=[1/2^{n-1}+\sum _{i=1}^{n-1}(2m+1)^{n-i}/2^i]^p$$ and $$S^\triangle _n(-1,x(-1))=\{(2m+1)^{np}-[1/2^{n-1}+\sum _{i=1}^{n-1}(2m+1)^{n-i}/2^i]^p]\}/2$$. Let $$a=-2$$ and $$b=2$$. Then $$x(t)\not \equiv 0$$ is a solution of (5) satisfying the anti-periodic boundary conditions (6). Thus we have
  $$\int ^2_{-2}|u(t)|^{\frac{p+1}{p}}\Delta t\ge \frac{2^{(n-1)(p+1)+1-\frac{1}{p}}}{\left[ \int ^2_{-2}\frac{\Delta t}{|t|^{\frac{2m}{p}}}\right] ^{p+1}\prod _{i=1}^{n-1}\left\{\prod _{j=1}^n\left[\int ^2_{-2} \frac{\Delta t}{|t|^{\alpha _i}}\right]^{\frac{1}{p_j}}\right\}^{p+1}}.$$

### *Example 2*

Suppose that $\alpha _i\in {\mathbf{R}}\,(1\le i\le n)$, $p_1=p+1$ and $p_j\in (1,+\infty )\,(2\le j\le n)$ with $\sum ^n_{i=1}\alpha _i/p_i=\sum ^n_{i=1}1/p_i=1$. Let ${\mathbf{T}}=\{\pm 2^n:n=0,1,2,\ldots \}$, $a_k(t)=t$ for $1\le k\le n-1$ and $a_n=t^{2}$ and $u(t)=-(\sigma (t)+t)/t^{p}$. Write $x(t)=t$. It is easy to check that $S_k(t,x(t))=t\,(0\le k\le n-1)$, $S_n(t,x(t))=t^2$ and $S^\triangle _n(t,x(t))=\sigma (t)+t$.

Let $a=-2^r$ and $b=2^r$ for some positive integer *r*. Then $x(t)\not \equiv 0$ is a solution of (5) satisfying the anti-periodic boundary conditions (6). Thus we have

$$\int ^{2^r}_{-2^r}|\frac{\sigma (t)+t}{t^{p}}|^{\frac{p+1}{p}}\Delta t\ge \frac{2^{(n-1)(p+1)+1-\frac{r}{p}}}{\left[ \int ^{2^r}_{-2^r}\frac{\Delta t}{|t|^{\frac{2}{p}}}\right] ^{p+1}\prod _{i=1}^{n-1}\left\{\prod _{j=1}^n\left[\int ^{2^r}_{-2^r} \frac{\Delta t}{|t|^{\alpha _i}}\right]^{\frac{1}{p_j}}\right\}^{p+1}}.$$

Now, we give an application of Lyapunov-type inequality of Theorem 4 for the following eigenvalue problem

14 $$S_n^\triangle (t,x(t))+ru(t)x^{p}(t)=0$$

on time scale $[a,b]_{\mathbf{T}}$ for some $a,b\in {\mathbf{T}}$ with $a<b$, where $S_0(t,x(t))=x(t), S_k(t,x(t))=a_k(t)S^\triangle _{k-1}(t,x(t))$ for $1\le k\le n-1$ and $S_n(t,x(t))=a_n(t)[S_{n-1}^\Delta (t,x(t))]^p$, $a_k\in C_{rd}([a,b]_{\mathbf{T}},(-\infty ,0)\cup (0,\infty ))\,(1\le k\le n)$ with $a_n(a)=a_n(b)$ and $u\in C_{rd}([a,b]_{\mathbf{T}}, {\mathbf{R}})$, *p* is the quotient of two odd positive integers. It is easy to see the lower bound of the eigenvalue *r* in (14)

$$|r| \ge \frac{2^{(n-1)p+1}}{\left[\int ^b_a|u(t)|^{\frac{p+1}{p}}\Delta t\right]^{\frac{p}{p+1}}(b-a)^{\frac{ 1}{p+1}}\left[ \int ^b_a\frac{\Delta t}{|a_n(t)|^{\frac{1}{p}}}\right] ^{p}\prod _{i=1}^{n-1}\left\{\prod _{j=1}^n\left[\int ^b_a \frac{\Delta t}{|a_i(t)|^{\alpha _i}}\right]^{\frac{1}{p_j}}\right\}^{p}},$$

where $\alpha _i\in {\mathbf{R}}\,(1\le i\le n)$, $p_1=p+1$ and $p_j\in (1,+\infty )\,(2\le j\le n)$ with $\sum ^n_{i=1}\alpha _i/p_i=\sum ^n_{i=1}1/p_i=1$.

## Conclusions

In this paper, we establish a Lyapunov-type inequality for the following higher order dynamic equation

$$S_n^\triangle (t,x(t))+u(t)x^{p}(t)=0$$

on some time scale **T** under the anti-periodic boundary conditions (6). Our results complement with some previous ones.
